# Potential Impacts of Climate Change on the Habitat Suitability of the Dominant Tree Species in Greece

**DOI:** 10.3390/plants11121616

**Published:** 2022-06-20

**Authors:** Nikolaos M. Fyllas, Theano Koufaki, Christodoulos I. Sazeides, Gavriil Spyroglou, Konstantinos Theodorou

**Affiliations:** 1 Biodiversity Conservation Laboratory, Department of Environment, University of the Aegean, 81100 Mytilene, Greece; koufaki@env.aegean.gr (T.K.); sazeides@env.aegean.gr (C.I.S.); ktheo@aegean.gr (K.T.); 2 Forest Research Institute, Hellenic Agricultural Organisation “Demeter”, 57006 Thessaloniki, Greece; spyroglou@fri.gr

**Keywords:** species distribution modelling, maximum entropy, range shifts, SSPs scenarios

## Abstract

Climate change is affecting species distribution and ecosystem form and function. Forests provide a range of ecosystem services, and understanding their vulnerability to climate change is important for designing effective adaptation strategies. Species Distribution Modelling (SDM) has been extensively used to derive habitat suitability maps under current conditions and project species distribution shifts under climate change. In this study, we model the current and future habitat suitability of the dominant tree species in Greece (*Abies cephalonica*, *Abies borisii-regis*, *Pinus brutia*, *Pinus halepensis*, *Pinus nigra*, *Quercus ilex*, *Quercus pubescens*, *Quercus frainetto* and *Fagus sylvatica*), based on species-specific presence data from the EU-Forest database, enhanced with data from Greece that is currently under-represented in terms of tree species occurrence points. By including these additional presence data, areas with relatively drier conditions for some of the study species were included in the SDM development, yielding a potentially lower vulnerability under climate change conditions. SDMs were developed for each taxon using climate and soil data at a resolution of ~1 km^2^. Model performance was assessed under current conditions and was found to adequately simulate potential distributions. Subsequently, the models were used to project the potential distribution of each species under the SSP1-2.6 and SSP5-8.5 scenarios for the 2041–2070 and 2071–2100 time periods. Under climate change scenarios, a reduction in habitat-suitable areas was predicted for most study species, with higher elevation taxa experiencing more pronounced potential habitat shrinkages. An exception was the endemic *A. cephalonica* and its sister species *A. borisii-regis*, which, although currently found at mid and high elevations, seem able to maintain their potential distribution under most climate change scenarios. Our findings suggest that climate change could significantly affect the distribution and dynamics of forest ecosystems in Greece, with important ecological, economic and social implications, and thus adequate mitigation measures should be implemented.

## 1. Introduction

In Europe, forests cover around 40% of the land, while in Greece, the latest estimates indicate that forests cover around 31.5% of the total land area [1]. Forests provide several ecosystem services, such as climate regulation, water supply, timber, energy, food and habitat for many species [2]. Particularly due to their ability to regulate local and global climate through carbon and water cycling, forest ecosystem function under global change conditions is of great interest for biodiversity conservation and climate adaptation planning [3].

The response of forests to environmental shifts is usually studied with field-based long-term measurements [4] and satellite image analysis [5], as well as with process-based models that simulate the response of species and communities to environmental variation [6]. At the European scale, simulations of future vegetation distribution suggest that at least 1/3 of the land surface area may be covered by different (to current) vegetation by the end of the century [7,8]. In southern Europe, shifts in the dominant vegetation type are expected to be even more pronounced, and widespread replacement of forest from shrubland has been predicted, primarily as an effect of drier conditions and interactions with fire [9,10]. In Greece, a process-based evaluation of how forest ecosystems will respond to climate change is available only for a limited number of sites [11,12]. These simulations are in line with the general trend of elevation shifts in species distribution and the replacement of drought-sensitive from drought-resistant species, with positive interactions with the local fire regime also identified [13]. 

Although local simulations from process-based ecosystem models are useful to understand the mechanisms of change in forest function, regional-scale projections under climatic change scenarios are also important, as they provide a wider overview of the expected changes. For that purpose, species distribution models (SDMs), or niche models, have been extensively used to model the potential distribution of both species and ecosystems [14,15]. A species niche is traditionally [16] defined as the “N-dimensional hypervolume where a species could persist”, and in the case of SDMs, the N-dimensions are represented by suites of environmental predictor variables. By modelling a species niche, SDMs help us to identify the key environmental factors that shape a species distribution [17]. In practice, SDMs combine current presence data with several environmental factors, such as climatic or edaphic, to simulate habitat suitability [14], although joint species distribution modelling has also been proposed [18]. After fitting an adequate model, the “function” describing species occurrence is used to simulate species distribution across a range of environmental conditions [15]. SDMs provide a quick and spatially explicit simulation of a species niche, and this is one of the reasons they have been extensively used to study how forests will respond to climate change [19,20]. 

The limitations of SDMs are also well documented and include: (a) uncertainties in environmental predictors and collinearity between them [21], (b) the in-built assumption that the relationship between species presence/absence and environmental predictors (parameterised on historical or current distribution data) will maintain under future conditions [22], (c) the fact that SDMs usually have no mechanistic basis and key plant processes related to species physiology and adaptation are not taken into account [23,24] and (d) that they are trained based on the realised (including the effect of competition and dispersal limitation) and not the fundamental (physiological) species niche, i.e., on a restricted environmental space [22]. The first limitation can be dealt with appropriate modelling techniques and improvements in the accuracy of databases used to train SDMs [25]. The second limitation is related to the fact that for species with long generation lengths, such as trees, adaptation cannot follow the pace of environmental change, and species response would almost solely come from phenotypic plasticity [26]. The third limitation could potentially be dealt with by combining empirical and process-based models [27], which was not, however, the purpose of this study. The fourth limitation has been challenged by Soberon and Peterson [28], suggesting that in many cases, SDMs provide an approximation of the fundamental niche. On the other hand, Araujo and Guisan [17] suggested that due to the practical difficulties in distinguishing between the fundamental and the realised niche, a possible solution for SDMs would be to disregard the two concepts and treat observed species distribution as an incomplete definition of the abiotic and biotic conditions that allow species to persist in the landscape. In that sense, as has been highlighted by Araujo and Guisan [17], it is important to treat spatial SDM projections as potential habitats for the species under study rather than their potential geographical distributions. 

**Figure A1:**
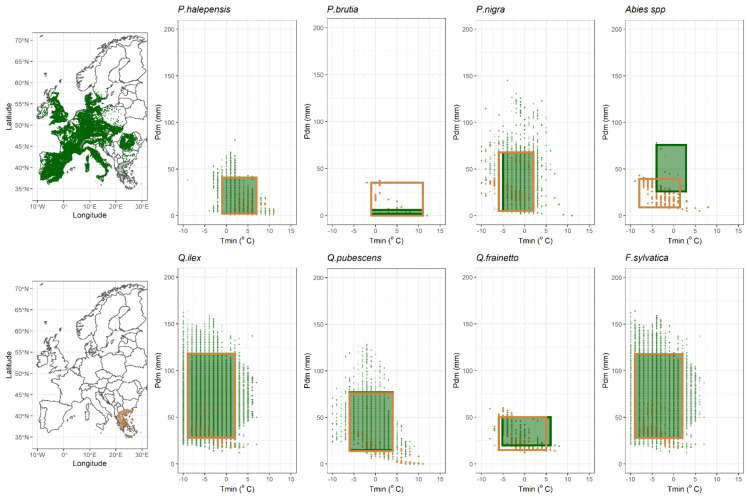
Two-dimensional bioclimatic envelopes of the studied species (green crosses and rectangles) as defined using the EU-Forest database [30], in contrast to the 2D envelope defined using the enhanced dataset with species presence in Greece (orange dots and rectangles). The limits of the rectangles are defined using the 0.025 and 0.975 quantiles for the mean daily minimum air temperature of the coldest month (Tmin) and the precipitation of the driest month (Pdm). For *P. brutia, P. nigra, Abies* spp. and *Q. frainetto,* the non-overlapping rectangles suggest that the species’ niche could have been extended in this study, including drier areas and decreasing the simulated species’ vulnerability to climate change. The study area is shaded in grey.

In this article, we model the distribution of the dominant tree species (*Abies cephalonica* Loudon and *Abies borisii-regis* Mattf treated as a single species, *Pinus halepensis* Miller, *Pinus brutia* Ten., *Pinus nigra* Arn., *Quercus ilex* L., *Quercus pubescens* Willd., *Quercus frainetto* Ten. and *Fagus sylvatica* L. *s.l.*) in Greece, under current and global warming conditions. Our choice was primarily motivated by the under-representation in the EU-Forest database [29,30] of species presence datapoints in Greece. SDMs are sensitive to sampling bias, as a result of different sampling efforts from one environmental context to another [31,32]. Moreover, when wide geographic areas (as in our case the Grecian peninsula) are systematically under-represented, the lack of presence data can introduce bias when predicting species habitat suitability due to the exclusion of sets of environmental conditions, which for some species represent the drier end of their distribution limit (Figure A1). As most of our study species can be found across the Grecian peninsula and its southernmost part, this could lead to an overestimation of their predicted vulnerability to climate change. We, therefore, increased the presence points of the study species in Greece, with our own observations and data from forest stewardship plans, from a few tens to a few thousand. Subsequently, we used Maxent, a widely used, highly performant SDM algorithm [33,34], to simulate the current habitat suitability of the study species in Greece, using a suite of climate and edaphic variables. We then combined the SDM models with projections from general circulation models for two contrasting climate change scenarios and time periods (2041–2070 and 2071–2100) to predict the range of change in (i) the surface of suitable habitat for each species, and (ii) the elevational shift in habitat suitability. Given the uncertainties and limitations in SDMs projections for future plant species distribution [35], we did not take these outputs for granted, and we thus discuss our findings in conjunction with results from dendroecological and ecophysiological studies in order to sketch the potential vulnerability of each species to the expected climatic shifts over the 21st century.

## 2. Results

### 2.1. Species Habitat Suitability under Current Conditions

**Figure 1:**
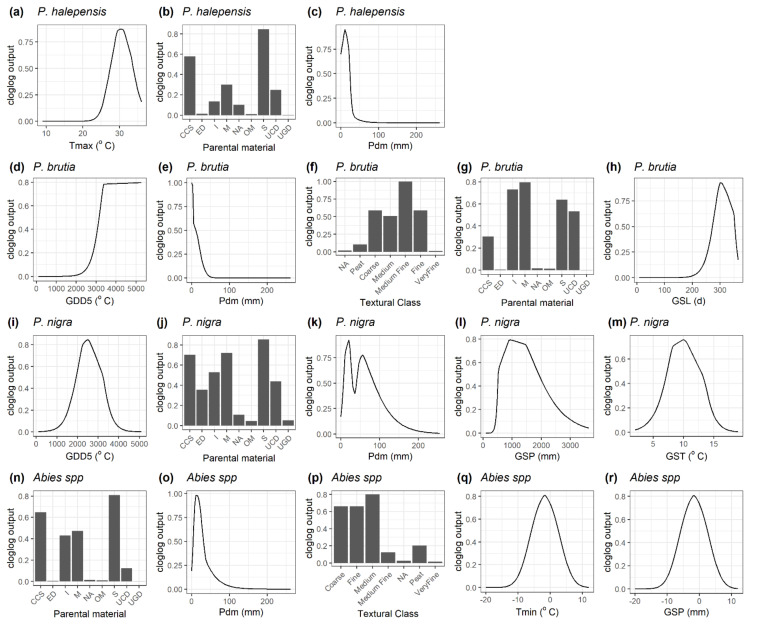
Response curves of the studied conifer species to each environmental variable maintained in the final maximum entropy model. Variables are sorted from left to right based on their relative contribution (decreasing order) (see also Table 1). T_max_: mean daily maximum air temperature of the warmest month (°C), T_min_: mean daily minimum air temperature of the coldest month (°C), P_a_: total annual precipitation (mm), P_dm_: precipitation of the driest month (mm), GDD5: heat sum of all days above 5 °C accumulated over a year (°C), GSL: length of the growing season (days), GST: mean temperature of all growing season days (°C), GSP: precipitation accumulated during the growing season (mm). Parental material classes include CCS: consolidated-clastic-sedimentary rocks, ED: eolian deposits, I: igneous rocks, M: metamorphic rocks, NA: no information, OM: organic materials, S: sedimentary rocks (chemically precipitated, evaporated or organogenic or biogenic in origin), UCD: unconsolidated deposits (alluvium, weathering residuum and slope deposits), UGD: unconsolidated glacial deposits/glacial drift. Texture (surface) dominant classes include NA: No information, Peat: No mineral texture: Coarse (18% < clay and >65% sand), Medium (18% < clay < 35% and ≥15% sand, or 18% < clay and 15% < sand < 65%), Medium fine (<35% clay and <15% sand), Fine (35% < clay < 60%), Very fine (clay > 60%).

**Figure 2:**
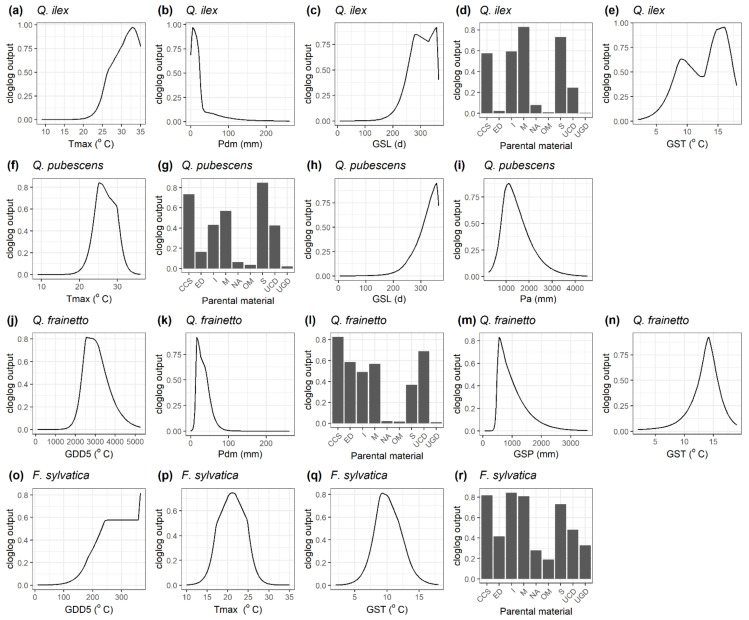
Response curves of the studied broadleaved species to each environmental variable maintained in the final maximum entropy model. Variables are sorted from left to right based on their relative contribution (decreasing order) (see also Table 1). T_max_: mean daily maximum air temperature of the warmest month (°C), T_min_: mean daily minimum air temperature of the coldest month (°C), P_a_: total annual precipitation (mm), P_dm_: precipitation of the driest month (mm), GDD5: heat sum of all days above 5 °C accumulated over a year (°C), GSL: length of the growing season (days), GST: mean temperature of all growing season days (°C), GSP: precipitation accumulated during the growing season (mm). Parental material classes include CCS: consolidated-clastic-sedimentary rocks, ED: eolian deposits, I: igneous rocks, M: metamorphic rocks, NA: no information, OM: organic materials, S: sedimentary rocks (chemically precipitated, evaporated or organogenic or biogenic in origin), UCD: unconsolidated deposits (alluvium, weathering residuum and slope deposits), UGD: unconsolidated glacial deposits/glacial drift. Texture (surface) dominant classes include NA: No information, Peat: No mineral texture: Coarse (18% < clay and >65% sand), Medium (18% < clay < 35% and ≥15% sand, or 18% < clay and 15% < sand < 65%), Medium fine (<35% clay and <15% sand), Fine (35% < clay < 60%), Very fine (clay > 60%).

**Figure A2:**
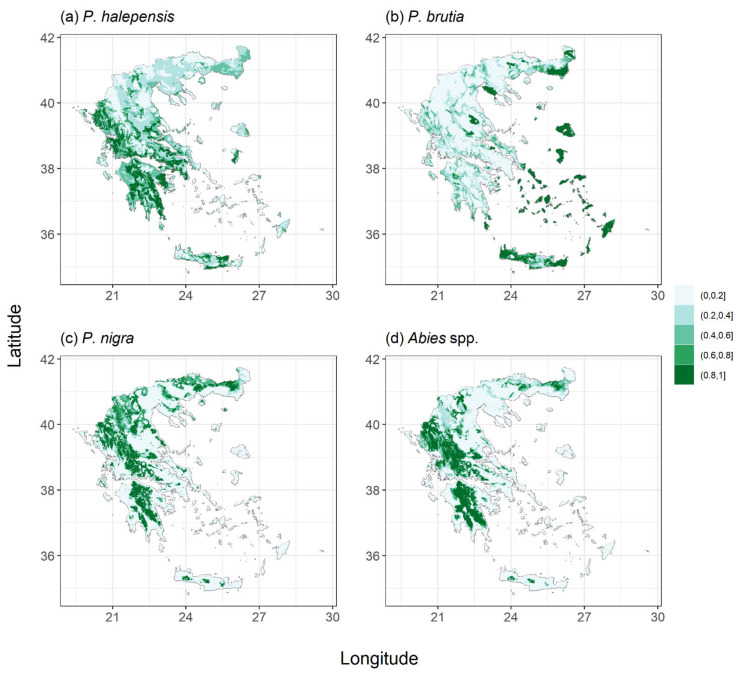
Habitat suitability maps of the studied conifer species under current climate conditions. The species-specific maps are also provided as GeoTIFF files in Supplementary File S1.

**Figure A3:**
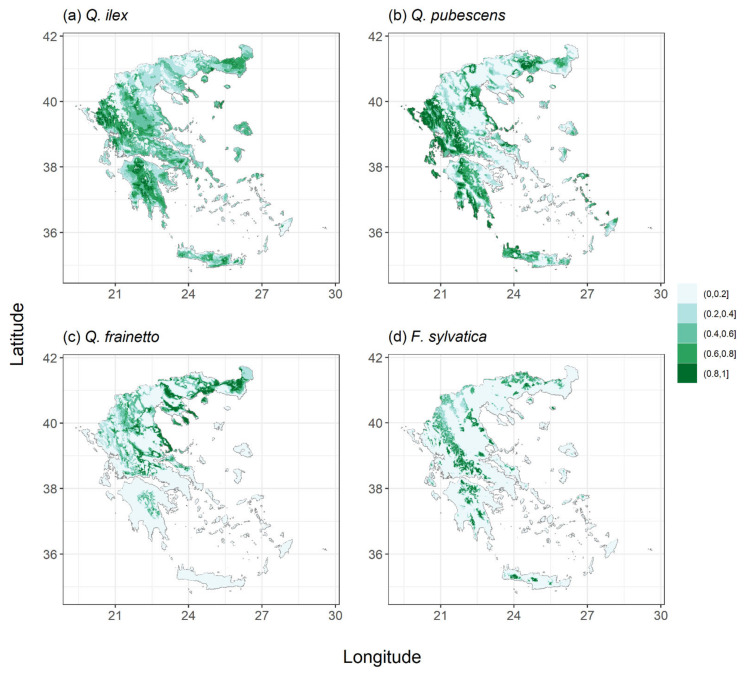
Habitat suitability maps of the studied broadleaf species under current climate conditions. The species-specific maps are also provided as GeoTIFF files in Supplementary File S1.

Under current climate conditions, the final models adequately simulated the distribution of all species, with an AUC ranging from 0.86 for *P. nigra* and *F. sylvatica* up to an AUC of 0.98 for *P. brutia*. Table 1 summarises the relative contribution of each environmental predictor to the distribution of the study species, with Figure 1 and Figure 2 illustrating the species-specific response curves. T_max_ strongly explained the habitat suitability of *P. halepensis* with an optimum of around 30 °C (Figure 1a), while the species was mainly found on sedimentary consolidated-clastic-sedimentary rocks (Figure 1b). The habitat suitability of *P. brutia* increased with the amount of GDD5 with an asymptote around 3500 °C (Figure 1d) and decreased with P_dm_ (Figure 1e). The species was more abundant in medium-fine soil texture class (Figure 1f) and igneous and metamorphic rocks (Figure 1g). *P. nigra’s* habitat suitability illustrated an optimum of GDD5 around 2500 °C (Figure 1i) and of GSP around 1000 mm (Figure 1l). The species was mainly found on the sedimentary and metamorphic parental material classes (Figure 1j). *A. cephalonica* and *A. borisii-regis* were mainly found in sedimentary and consolidated-clastic-sedimentary rocks (Figure 1n) and medium textural classes (Figure 1p). Dry month precipitation contributed to the habitat suitability of *Abies* spp. with an optimum of around 25 mm (Figure 1o). *Q. ilex* habitat suitability indicated an optimum T_max_ of around 32 °C (Figure 2a) and decreased with P_dm_ (Figure 2b). The species’ presence was strongly related to metamorphic and sedimentary parental material (Figure 2d). *Q. pubescens* suitability indicated an optimum T_max_ of around 26 °C (Figure 2f) and P_a_ around 1000 mm (Figure 2i), while it was mainly associated with consolidated-clastic-sedimentary and sedimentary rocks (Figure 2g). The habitat suitability of *Q. frainetto* was strongly associated with GDD5 with an optimum between 2500 and 3000 °C (Figure 2j) and decreased with a P_dm_ above 50 mm (Figure 2k). Finally, the habitat suitability of *F. sylvatica* increased with GSL (Figure 2o) with a T_max_ optimum of around 22 °C (Figure 2p) and GST between 9 and 11 °C (Figure 2q). The species-specific Maxent models were subsequently used to predict the current habitat suitability for the study species along the Grecian peninsula (Figure A2 and Figure A3, Supplementary File S1). 

### 2.2. Species Habitat Suitability under Climate Change

**Figure 3:**
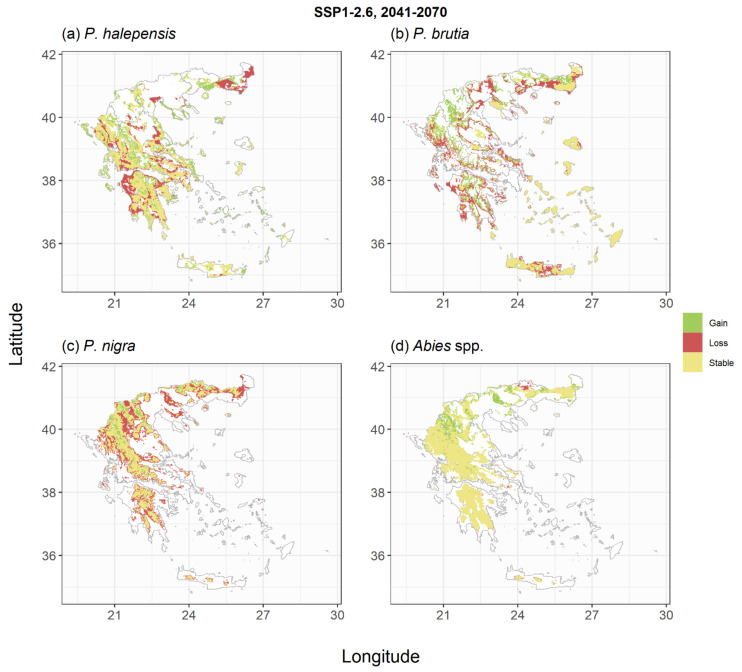
Shifts in habitat suitability of the studied conifer species under the SSP1-2.6 climate scenario for the period 2041–2070. Areas with green indicate regions that the species could expand, areas with yellow indicate regions that the species could have similar to current environmental conditions and areas with red indicate regions that would not be suitable under future conditions. The scenario- and species-specific maps are provided as GeoTIFF files in Supplementary File S2.

**Figure 4:**
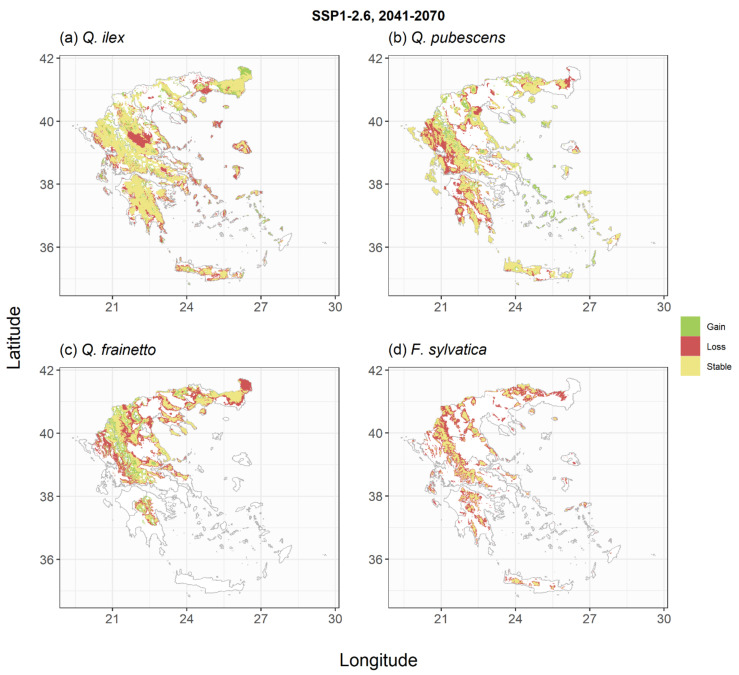
Shifts in habitat suitability of the studied broadleaf species under the SSP1-2.6 climate scenario for the period 2041–2070. Areas with green indicate regions that the species could expand, areas with yellow indicate regions that the species could have similar to current environmental conditions and areas with red indicate regions that would not be suitable under future conditions. The scenario- and species-specific maps are provided as GeoTIFF files in Supplementary File S2.

**Figure 5:**
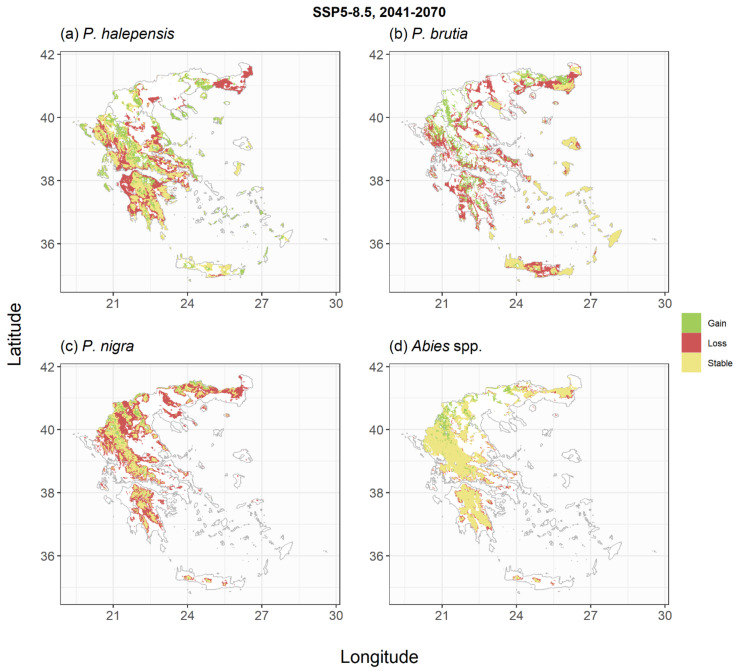
Shifts in habitat suitability of the studied conifer species under the SSP5-8.5 climate scenario for the period 2041–2070. Areas with green indicate regions that the species could expand, areas with yellow indicate regions that the species could have similar to current environmental conditions and areas with red indicate regions that would not be suitable under future conditions. The scenario- and species-specific maps are provided as GeoTIFF files in Supplementary File S3.

**Figure 6:**
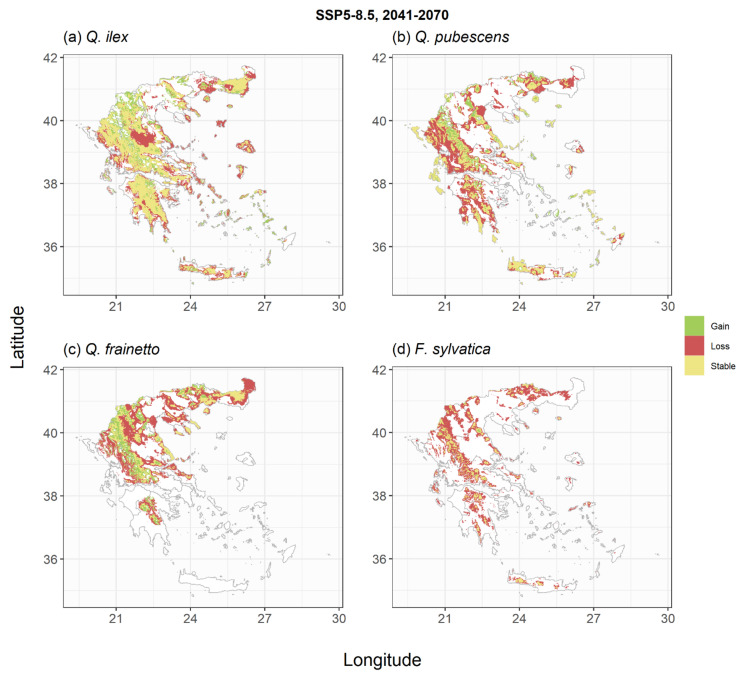
Shifts in habitat suitability of the studied broadleaf species under the SSP5-8.5 climate scenario for the period 2041–2070. Areas with green indicate regions that the species could expand, areas with yellow indicate regions that the species could have similar to current environmental conditions and areas with red indicate regions that would not be suitable under future conditions. The scenario- and species-specific maps are provided as GeoTIFF files in Supplementary File S3.

**Figure A4:**
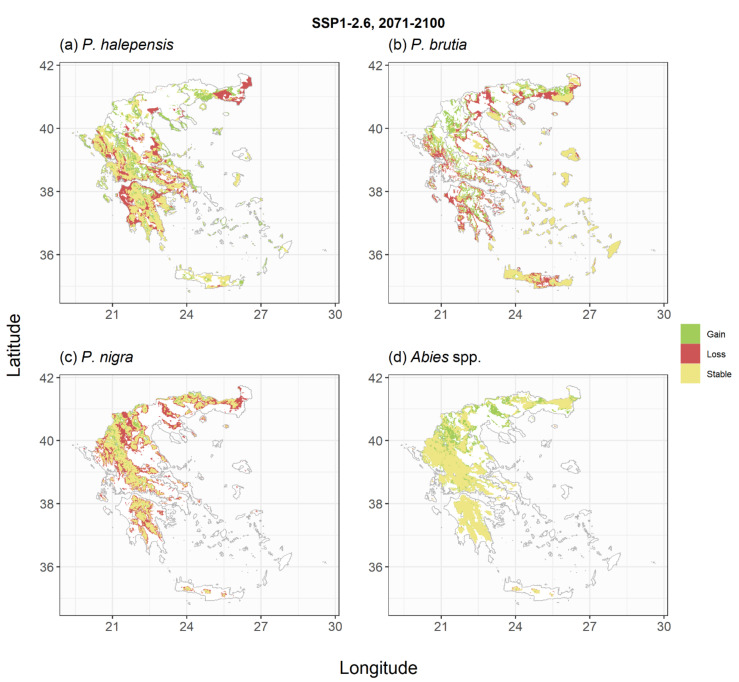
Shifts in habitat suitability of the studied conifer species under the SSP1-2.6 climate scenario for the period 2071–2100. Areas with green indicate regions that the species could expand, areas with yellow indicate regions that the species could have similar to current environmental conditions and areas with red indicate regions that would not be suitable under future conditions. The scenario- and species-specific maps are also provided as GeoTIFF files in Supplementary File S4.

**Figure A5:**
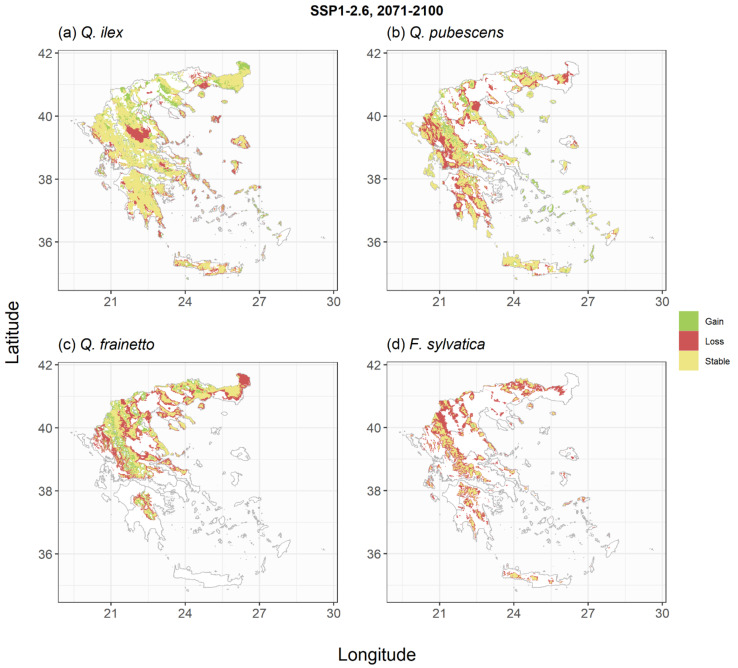
Shifts in habitat suitability of the studied broadleaf species under the SSP1-2.6 climate scenario for the period 2071–2100. Areas with green indicate regions that the species could expand, areas with yellow indicate regions that the species could have similar to current environmental conditions and areas with red indicate regions that would not be suitable under future conditions. The scenario- and species-specific maps are also provided as GeoTIFF files in Supplementary File S4.

**Figure A6:**
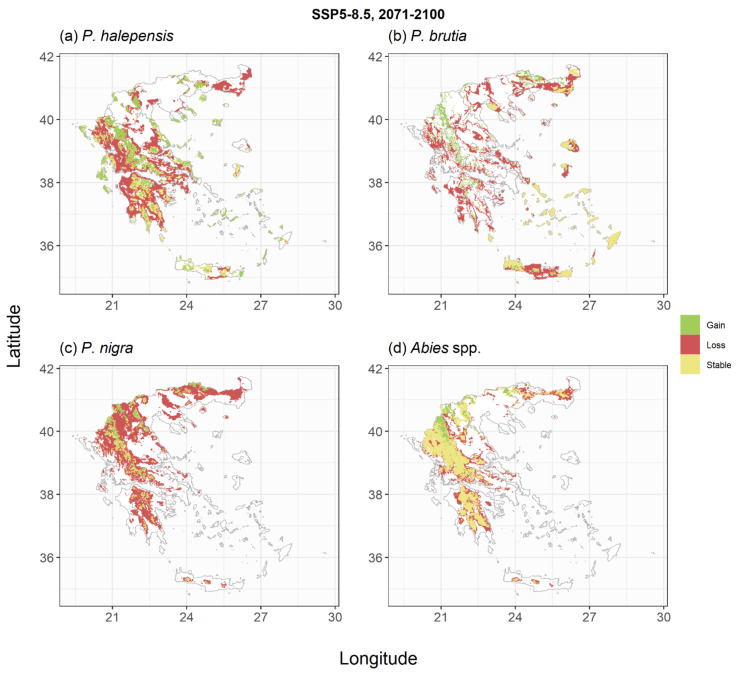
Shifts in habitat suitability of the studied conifer species under the SSP5-8.5 climate scenario for the period 2071–2100. Areas with green indicate regions that the species could expand, areas with yellow indicate regions that the species could have similar to current environmental conditions and areas with red indicate regions that would not be suitable under future conditions. The scenario- and species-specific maps are also provided as GeoTIFF files in Supplementary File S5.

**Figure A7:**
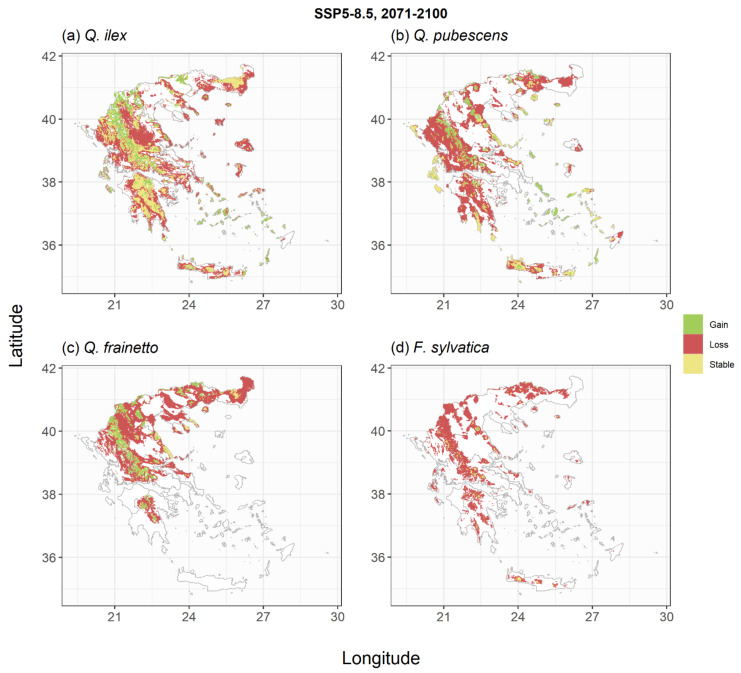
Shifts in habitat suitability of the studied broadleaf species under the SSP5-8.5 climate scenario for the period 2071–2100. Areas with green indicate regions that the species could expand, areas with yellow indicate regions that the species could have similar to current environmental conditions and areas with red indicate regions that would not be suitable under future conditions. The scenario- and species-specific maps are also provided as GeoTIFF files in Supplementary File S5.

The trained models were subsequently used to derive species habitat suitability under the two climate change scenarios and two periods of interest (Figure 3, Figure 4, Figure 5, Figure 6, Figure A4, Figure A5, Figure A6 and Figure A7). For *P. halepensis,* a relatively small reduction in suitable areas from −6% to −8% was projected under the SSP1-2.6 scenarios (Table 2, Figure 3a and Figure A4a), which increased up to 21–45% under the extreme SSP5-8.5 scenarios (Figure 5a and Figure A6a). These reductions were associated with a mean elevation shift, ranging from +139 to +330 m, compared to the current species distribution (Table 2). *P. brutia* was projected to suffer relatively higher habitat area losses, ranging between −14% and −17% for the two reference periods of the SSP1-2.6 scenario, up to 32–54% under the extreme SSP5-8.5 scenario. These reductions were followed by a mean elevation shift, ranging from +164 to +333 m, compared to the current species distribution (Table 2). An extensive reduction of suitable habitat for *P. nigra* was projected under all climate change scenarios (Figure 3c, Figure 5c, Figure A4c and Figure A6c), with the SSP5-8.5 projection for the 2100 period yielding a reduction up to 77% compared to current climate conditions (Table 2), and an average elevation shift of up to +599 m. On the other hand, projections for the two *Abies* species suggest that at least following the mild SSP1-2.6 scenarios, these species could even increase their potential distribution area by +17% and +25% in the 2041–2070 (Figure 3d) and the 2071–2100 periods (Figure A4d), respectively. However, under the extreme SSP5-8.5 scenario, they were projected to either maintain their total suitable area (Figure 5d) in the short term or shrink by −27% in the longer-term (Figure A6d). The low elevation holm oak (*Q. ilex*) was projected to suffer small area losses (from −1% to −14%) following the mild SSP1-2.6 scenarios (Figure 4a and Figure A5a), and more extended area losses (from −18% to −47%) under the extreme SSP5-8.5 scenarios (Figure 6a and Figure A7a), associated with mean elevation shifts from +71 to +387 m, respectively. Simulations of the *Q. pubescens* habitat suitability suggested a decrease in suitable areas (from −16% to −64%, Figure 4b, Figure 6b, Figure A5b and Figure A7b), accompanied by elevational shifts from +143 to +306 m. This was also the case for *Q. frainetto,* with more widespread losses (from −28% to −72%, Figure 4c, Figure 6c, Figure A5c and Figure A7c) and greater mean elevation shifts (from +233 to +650 m). Potential area losses were even more pronounced for *F. sylvativa* ranging between −56% for the short term SSP1-2.6 scenario and up to −93% under the extreme SSP5-8.5 long-term scenario (Figure 4d, Figure 6d, Figure A5d and Figure A7d). 

## 3. Discussion

**Figure A8:**
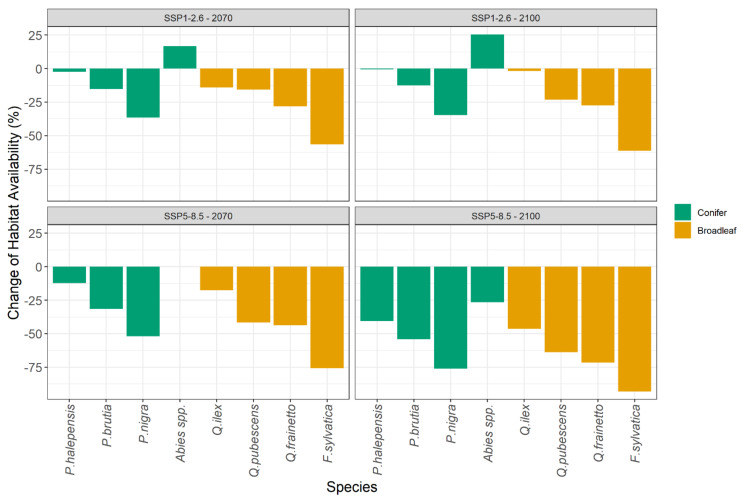
Barplot of changes (%) in habitat availability of the study species under the two climate change scenarios and reference periods. Species are grouped to conifers (green) and broadleaved (orange) and sorted based on their current elevation range of expansion from lower to higher elevations.

Our findings suggest that climate change might lead to significant shifts in the habitat suitability of the dominant forest tree species in Greece. Overall, the thermomediterranean and more drought-resistant *P. brutia*, *P. halepensis* and *Q. ilex* are projected to suffer smaller suitable habitat area losses compared to meso- and supra-mediterranean elevation species, such as *P. nigra*, *Q. pubescens*, *Q. frainetto* and *F. sylvatica* (Figure A8). Of interest are the rather stable, under most scenarios, projections for the two *Abies* species. The selected study species represent key elements of forest ecosystems in Greece, and our findings might be useful for inferring the vulnerability of the sector and its potential for adaptation under warmer future conditions. In the following paragraphs, we discuss our findings in conjunction with results from other habitat suitability studies at the European scale, as well as with dendroecological and ecophysiological studies and simulations based on process-based models.

SDMs have been frequently used to model the effects of climate change on the distribution of European tree species [36]. In general, some species can be grouped as “winners”, i.e., species that expand their distribution area or “losers”, i.e., species with projected shrinkages in their habitat suitability [37], although distinct geographical patterns are identified with mountainous Mediterranean tree species and rear edge temperate tree species populations projected to suffer habitat area losses [9,38,39]. Typical Mediterranean forest tree species (such as *P. halepensis*, *P. brutia* and *Q. ilex*) are projected to suffer small reductions in habitat suitability [38] or expand northwards [40,41], in agreement to our findings (Table 2). Studies on mountainous Mediterranean taxa, such as *P. nigra*, present contrasting findings, with either projected area expansions in Italy [42] or substantial losses in Turkey [43], with our findings supporting the second case. *Abies alba* has been projected to maintain [40] or to even expand its habitat suitability [37] across Europe, with our findings suggesting that the drought-resistant sister species of *A. cephalonica* and *A. borissi-regis* in Greece [44] could maintain their habitat-suitable areas under most climate change scenarios. Although across Europe, *Q. pubescens* is projected to enhance its habitat-suitable areas [40], simulations in Italy suggest a contraction of the species’ habitat suitability [42] in accordance with our findings. Finally, although across Europe, climate change projections with SDM suggest that *F. sylvatica* will remain rather stable [40] or even increase its habitat suitability [37], southern populations are expected to lose parts of their current suitable areas [38,45], in accordance with our projections for Greece.

From the study species, the two thermomediterranean (low elevation) pines showed a relatively small shrinkage of suitable habitat areas across both climate change scenarios and reference periods. The simulated rather small increase in the average elevation of the species potential distribution (from +22 to +115 m asl under the extreme SSP5-8.5 scenario during the 2071–2100 period) suggests that the bioclimatic conditions would remain relatively favourable for *P. halepensis* and *P. brutia* at low elevations (Table 2). Both species are considered drought resistant [46], with *P. halepensis* exhibiting a strong capacity for ecophysiological adjustment of traits such as water use efficiency that could help the species to maintain viable populations under warmer and drier conditions [47]. Across Greece, meso- and supra-mediterranean zones just above the current area of expansion of *P. halepensis* and *P. brutia* are frequently dominated by broadleaved species, which might experience increased drought stress under warmer conditions. *P. halepensis* and *P. brutia* could potentially inhabit such areas, where they are currently outcompeted by less drought-resistant species, particularly in cases where increased fire frequency could favour the two serotinous pine species [48,49]. On the other hand, dendroecological analyses in Greece highlight the dependence of both pine species’ growth on long-term water availability [50,51,52], in accordance with their P_dm_ response curve, while a higher temperature could lead to growth reductions and increased mortality [53], particularly of small-size trees [54]. Simulations with process-based models suggest that *P. halepensis* stands could increase their primary productivity under warmer and CO_2_-rich conditions at sites with adequate rainfall [27], but could only maintain very slow aboveground biomass increments at dry sites [55]. In addition to the above, stands dominated by these two pine species are more flammable compared to other taxa [56], and thus in conjunction with the expected expansion of the fire-risk period in Mediterranean regions [57], climate-driven vegetation shifts might lead to positive biotic feedbacks [13] that extent fire-prone areas. Overall, the integration of the published results with the projections of this study suggests that both *P. halepensis* and *P. brutia* will probably remain a key element of low-elevation forests in Greece during the 21st century in areas that will not experience an extensive reduction in water availability and increase in fire frequency. 

*Pinus nigra* can grow in a wide range of temperature and water availability conditions [58], but many studies highlight that this species might be sensitive to prolonged drought, both in terms of regeneration vigour [59,60] and growth [61,62]. Our projections suggest a strong shrinkage of the species’ habitat suitability, which, under the extreme SSP5-8.5 scenario, can reach up to 75% of its current extent (Table 2). Ecophysiological studies have found that *P. nigra* is relatively vulnerable to drought [63,64], with growth declines and diebacks reported, particularly in southern populations [65,66,67]. Empirical models suggest that under climate change, the growth of *P. nigra* in the Iberian Peninsula is expected to decrease, except for northern and productive areas [68,69]. Considering our projections and results from ecophysiological and dendroecological studies, it seems that under climate change, *P. nigra* forests in Greece would suffer significant area losses due to bioclimatic limitation, which could be further enhanced by the intensification of fire regimes in mountainous areas [70].

Our simulations project that habitat-suitable areas for the endemic Greek fir *A. cephalonica* and its *A. borisii-regis* hybrid are expected to remain rather stable or even increase under the mild SSP1-2.6 scenario (Table 2). *A. cephalonica* is known to follow a drought avoidance strategy that, by regulating stomata, achieves the highest water use efficiency among circum-Mediterranean firs [71,72]. At the same time, the temperature range that Greek fir populations are found in is wider than both other Mediterranean fir species as well as other typical mountainous species, such as *Q. frainetto, P. nigra* and *F. sylvatica* [72], suggesting a relative higher adaptability of the species to drought conditions. This could probably be the reason we found no association between T_max_ and habitat suitability in our simulations. However, *A. cephalonica* has experienced extensive dieback during severe drought years in the past [73,74], associated with water stress and/or insect outbreaks [75,76]. At the same time, dendroecological analyses have identified that the growth of *A. cephalonica* is positively related to spring and summer-time precipitation [42,77]. Although Koutavas [78] reports a growth acceleration of the species since the 1990s, potentially related to CO_2_ fertilisation, simulations with vegetation dynamics models (that did not, however, account for CO_2_ fertilisation) suggest that under drier conditions, *A. cephalonica* might be replaced by more drought-resistant species [12]. Other studies have shown that *A. cephalonica* regeneration is controlled by regional climatic conditions [79], fire [80] and the distance from unburned patches [76], suggesting that under more fire-prone conditions, the species might fail to regenerate. Overall, our findings, combined with previous work, suggest that the vulnerability of the endemic *A. cephalonica* to climate change could be highly regulated by local weather conditions and the interplay with fire regimes. 

The three oak species of our study represent a continuum of drought resistance within the *Quercus* genus [81]. *Q. ilex* is an evergreen oak species that grows on various soil types, and it is adapted to dry Mediterranean conditions, although less resistant to water stress compared to other evergreen species, such as *Quercus coccifera* [82,83]. *Q. pubescens* and *Q. frainetto* are two of the most common oak species in Greece, currently found at low to mid and mid-high elevations. *Q. pubescens* is a semi-deciduous species that can withstand water limitation, with recent studies showing that it can be as resistant to drought stress as evergreen oaks such as *Quercus ilex* [84]. *Q. frainetto*, on the other hand, seems to be less tolerant to drought compared with the two other oaks in the study [85]. This ecophysiological knowledge seems to agree with the projected relative habitat loss in our study (Table 2). In particular, the more drought-tolerant holm oak is projected to suffer lower area losses under all climate change scenarios. Dendroecological studies suggest that *Q. frainetto* growth is positively related to summer-time precipitation [52], showing an increased limitation due to drier conditions since the 1990s [86]. At the same time, drought seems to also increase the species mortality [87], at least in the southern range of the species expansion. Similarly, *Q. pubescens* growth and mortality are drought-sensitive and could trigger future forest declines [88]. Thus, for the three oak species under study, a reduction of habitat suitability is projected, with the thermomediterranean and more drought-resistant *Q. ilex* expected to experience less pronounced declines (Table 2).

*F. sylvatica* is one of the most abundant broadleaved species across Europe, and it is extensively used in forest transition strategies [89]. However, the species is known to be sensitive to low water availability and drought [89,90,91], but see [92], with its long-term growth in Greece positively related to summer water availability [52]. Currently, *F. sylvatica* suffers extensive growth reductions in large parts of Europe [93], with climate-related forest declines in the southern parts of its expansion also documented [94]. Empirical models based on dendroecological data project severe growth declines during the 21st century [95]. Our projections predict a strong decrease in beech habitat suitability under both climate scenarios, accompanied by a strong elevation shift, in agreement with other models that suggest that the species might expand its northern edge and lose habitat at the southern edge of its distribution under a warmer and drier climate [96]. Thus, although an increase in the growing period of *F. sylvatica* could lead to sustained productivity [55], potential water limitation could increase the species’ vulnerability and, in agreement with our findings, reduce beech distribution in Greece (Table 2).

Our study provides the most complete overview of the potential habitat suitability shifts of the important forest tree species in Greece. Most of our findings are in accordance with other modelling and ecophysiological studies, suggesting a higher vulnerability of mountainous tree species to climate change. 

## 4. Materials and Methods

### 4.1. Study Species

In Greece, almost half of the forest areas are currently managed (51.6%), with the other half dominated by evergreen sclerophyllous taxa. In this study, we focused on the distribution of nine dominant trees species in Greece: Aleppo pine (*Pinus halepensis* Mill.), brutia pine (*Pinus brutia* Ten.), black pine (*Pinus nigra* Arn.), Greek fir (*Abies cephalonica* L.) and its closely related hybrid (*Abies borisii-regis* Mattf), holm oak (*Quercus ilex* L.), downy oak (*Quercus pubescens* Willd.), Hungarian oak (*Quercus frainetto* Ten) and beech (*Fagus sylvatica* sl L.), which dominate the managed forest areas. Conifer forests cover around 42.6% of the total managed forest area, with 57.4% covered by broadleaved species [97]. Thermophilus low-elevation pines (*P. halepensis, P. brutia*) cover around 16.2% of the managed forest area, with a very low contribution of *Pinus pinea*. Mid to high elevation *P. nigra* stands cover approximately 8.4% and firs (mainly *A. cephalonica* and *A. borisii-regis*) around 16.2% of the managed forest area. *Q. ilex* is mainly found in unmanaged areas. Oak forests (mainly *Q. frainetto*, *Q. pubescens*, *Q. cerris* and *Q. robur*) cover around 43.8% of the managed forest areas, with *Q. frainetto* as the dominant species in around 80% of all oak forests [98,99]. In Greece, three *Fagus* subspecies are found, i.e., *F. sylvatica*, *F. orientalis* and *F. moesiaca*. In this study, they were treated as a single species with similar ecological characteristics, covering around 10% of the managed forest area. Overall, the forest tree species included in this study cover around 95.3% of the total forest area under management in Greece. 

Aleppo pine forests form scattered pure stands, regardless of the soil substrate, in mainland Greece, the lowlands and along the coasts of the Aegean and Ionian Seas, in Euboia and in the islands of Sporades. In Crete, the islands of the eastern Aegean and Thrace, Aleppo pine is replaced by brutia pine, which has similar stand and site requirements. *P. halepensis* and *P. brutia* are drought-resistant pines and have similar ecological characteristics [100]. However, *P. halepensis* is considered more drought-resistant than *P. brutia* [101]. These pine forests are often open, with a bushy understorey, composed of evergreen broadleaved “maquis” species. In Greece, the two pine species are dominant in low elevations (up to 800 m asl), and their wood is mainly used for fuel. The black pine (*P. nigra*) is one of the most important forest species from an economic and ecological point of view. *Pinus nigra* forests represent a European priority habitat type included in Annex I of the Council Directive 92/43/EEC on the conservation of natural habitats and of wild fauna and flora. It has a wide distribution on the mainland from Taygetos (southern Greece) to Evros (northern Greece), and is also abundant on the islands of Lesvos, Samos and Thassos. It forms pure and mixed stands in the para-Mediterranean vegetation zone with its optimum between 800 and 1500 m asl. It grows very well on poor, calcareous-dolomitic soils, as well as on ophiolite-serpentinite soils with toxic concentrations of metals (Mg, Al, Ni, Cr, etc.). The Greek fir (*A. cephalonica*) is an endemic species well adapted to even poor calcareous-limestone soils, and forms forests that spread throughout the high mountains (800–1700 m asl) of Central Greece and the Peloponnese [102], replacing deciduous oak forests at a higher elevation. King Boris fir (*A. borissi-regis*) is a hybrid between *A. cephalonica* and sliver fir (*A. alba*), and it is found in the northern part of Greece and the mountains of the Balkan peninsula between 800 and 1800 m asl. Despite the relatively low productivity fir stands in Greece, their wood is used in construction works and ship-yarding.

Holm oak (*Q. ilex*) is a shade and drought-tolerant evergreen oak. In Greece, holm oak can be found from sea level to 1000 m asl, depending on the orientation and the slope of the stands, and it is mainly used as fuelwood and for charcoal production. Downy oak (*Q. pubescens*) is a common oak species with a wide distribution in the continental and insular parts of Greece. It grows sporadically in pure stands, but primarily, it is mixed with other oak species, especially Hungarian oak (*Q. frainetto*) and other deciduous broad-leaved species of the submontane zone usually up to an elevation of 900–1200 m asl. Its wood is mainly used for fuel and charcoal production due to its high wood density. The Hungarian oak is the most common oak species in mainland Greece, dominating around 80% of all oak forests and covering 33% of all forested areas [98,99]. It grows in submontane and mountainous areas between 300 and 1200 m asl, with a distribution range starting from the Peloponnese (Parnonas, southern Greece). It occupies almost exclusively siliceous soils, more or less heavy and unsuitable for agriculture. It often forms pure stands and is sometimes mixed with other oaks and thermophilic deciduous trees. Traditionally, stands of the three oak species have been managed as coppice forests mainly for firewood production and only occasionally for lumber. Currently, a large part of these forests is under conversion into high forests for increased lumber production and better climate change mitigation. *Fagus sylvatica* s.l. is a shade-tolerant species located mostly at the mountainous sites of Greece at mid to high altitudes (600–1900 m asl). The species is mainly used for lumber as well for fuel wood

### 4.2. Species Presence Database

Detailed tree species occurrence data are lacking in Greece due to the incomplete National Forest Inventory. For that reason, we used the EU-Forest high-resolution tree occurrence dataset for Europe [30], which is a dataset of species presence that harmonises forest plot surveys from the National Forest Inventories at a 1 × 1 km grid. Although the EU-Forest dataset contains more than 91,000 presence points for the study species across Europe, there were only 60 records within Greece. For this reason, we extended the dataset with our own observations and data from forest stewardship plans in Greece. We specifically have added 209 *A. cephalonica*, 248 *A. borisii-regis*, 128 *P. halepensis,* 105 *P. brutia*, 431 *P. nigra*, 391 *Q. frainetto*, 194 *Q. pubescens* and 417 *F. sylvatica* occurrences. Thus, the extended dataset contained 93,319 presence points for the species of interest. We note that for some studied species, the additional presence points were found at the drier end of their current distribution and thus increased the simulated bioclimatic envelope, particularly for *A. cephalonica & A. borisii-regis*, *P. brutia* and *Q. frainetto* (Figure A1).

### 4.3. Climate and Soil Data

Climate data for both current (1981–2010) and future conditions were downloaded from the CHELSA Project [103] with a spatial resolution of around 1 km^2^. The climate variables of interest included the mean daily maximum air temperature of the warmest month (T_max_), the mean daily minimum air temperature of the coldest month (T_min_), the annual precipitation (P_a_), the precipitation of the driest month (P_dm_), the heat sum of all days above 5 °C accumulated over a year (GDD5), the length of the growing season (GSL), the precipitation accumulated during the growing season (GSP) and the mean temperature of all growing season days (GST). Those eight climate variables were selected to represent four ecological dimensions considered important for tree species distribution: temperature stress (T_max_, T_min_), water availability/stress (P_a_, P_dm_), growing season length (GDD5, GSL) and growing season “quality” (GSP, GST). The first two dimensions, i.e., temperature and water stress, are considered important for controlling the distribution of typical Mediterranean species [104,105], while the latter two, i.e., growing season length and “quality”, are considered important for predicting the treeline [106]. The same climate variables were used to simulate the species’ future distribution based on projections of the Coupled Model Intercomparison Project Phase 6 (CMIP6) [107] from the Geophysical Fluid Dynamics Laboratory Earth System Model (GFDL-ESM4) [108] for a low and a high-carbon emissions scenario (Shared Socio-economic Pathways (SSPs): 1–2.6 and 5–8.5), for the time periods 2041–2070 and 2071–2100. The downscaling of GFDL-ESM4 outputs to 1 km^2^ has been made within the CHELSA database using statistical downscaling of atmospheric temperature and an algorithm that incorporates orographic predictors for precipitation [103]. 

Soil data were extracted from the European Soil Data Centre (ESDC) at a resolution of 1 km^2^ [109]. In this analysis, we extracted two categorical edaphic variables, i.e., the dominant parent material (parmat) and soil texture class (texture) expressing water and nutrients availability. 

### 4.4. Species Distribution Modelling

The extended dataset of species occurrence was thinned to address issues with spatial sampling biases using the *spThin* package [110]. In particular, the *thin* function takes a set of occurrence records and identifies multiple random new subsets that meet a minimum nearest neighbour distance constrain. From the new subsets, the one with the largest number of records was maintained. The default 10 km distance between presence points with 10 random repetitions was used for each species. The number of thinned species-specific occurrences ranged from 38 points for *P. brutia* to 6634 points for *F. sylvatica* (Table 1). We note that although we were interested in modelling the studied species’ habitat suitability in Greece, we used presence points in the European geographical range to train the SDMs. This was done because the outcome of the model can change according to the geographic extent used to train the model [111]. This is important to make sure that the breadth of the climatic conditions used in our models captured the full climatic niche of the species [112]. 

We used the maximum-entropy algorithm (MaxEnt) species distribution modelling algorithm [113] to predict the current and future habitat suitability of the study species. The MaxEnt is a machine learning method that estimates the suitability of an area by calculating the probability distribution of maximum entropy. It has been extensively used in a wide range of ecological applications (e.g., [34,114,115]) because it is one of the best-performing algorithms in species distribution modelling [116,117,118]. In particular, it has been proven useful for predicting the habitat suitability of tree species under current and future climate conditions (for example, [37,119]). Among its advantages is: (a) the fact that it requires presence-only data—as well as information about the external environment, usually referred to as background—without the need to explicitly define absence data; this is a very important feature of the method as absent data are notoriously difficult to obtain, (b) Maxent can be used with both continuous and categorical predictor variables and (c) its output, i.e., maximum likelihood estimate of the relative probability of presence, is continuous and easily interpretable. We implemented Maxent in R by using the package *SDMtune*, which, in addition to other features, includes data-driven variable selection algorithms [120]. 

These species-specific datasets were split into two parts, with 80% of the points used to train the model and 20% of the points to evaluate it. To run the models, background points were selected randomly, avoiding presence points [121], with their number being 3 times more than the occurrences present for each species. The model’s performance was evaluated with the AUC criterion (Area Under the Receiver Operating Characteristic (ROC) Curve). AUC quantifies the probability that the model correctly ranks a random presence locality higher than a random background pixel [113]. AUC ranges between 0 and 1, with higher values indicating a better model performance, while values < 0.5 show that the model is no better than random.

For each species, an initial model was created using the ten environmental predictors (eight climatic and two edaphic). A data-driven variable selection algorithm was implemented (function *varSel*) by iterating all variables in the order of their per cent contribution, identifying if they were highly correlated (Spearman’s r > 0.7) with any other predictor, running a leave one out Jackknife test and removing the variable that decreased the model performance the least when removed based on the AUC metric. A further simplification was implemented (function *reduceVar)* to remove variables that contributed less than 5 per cent to the model performance. For the final model, we assessed (i) the relative importance of environmental variables in determining habitat suitability using their permutation importance and (ii) the response curve, i.e., the relationship between habitat suitability and each predictor variable. To calculate the response curves, the response is modelled for one predictor variable while the other variables are held constant at their mean. 

The final models were then used to project each species’ distribution across Greece under current and future climate conditions (SSPs: 1.2–6 and 5.8–5 for 2041–2070 and 2071–2100). The predictions were cropped to the extent of the Greek territory to acquire habitat suitability maps of the current and future distribution of each species. These maps have continuous values ranging from 0 (unsuitable) to 1 (optimal) using the complementary log–log transform (cloglog) [122]. We, moreover, transformed the continuous suitability probability to a dichotomous suitable–unsuitable variable by applying a cut-off threshold based on the average predicted probability/suitability of each species occurrence. We adopted this method for two reasons: first, it has been shown to perform equally well as other widely used methods, such as the sensitivity and specificity combined approaches [123], and second, it maximized the agreement between the observed and predicted distributions for all species. The maps produced using the suitable–unsuitable approach classify the habitat under future conditions into three categories: (i) suitable under both current and future conditions (stable), (ii) suitable under current conditions but not under future ones (loss), and (iii) unsuitable under current conditions but predicted to become suitable in the future (gain). 

Furthermore, to evaluate the degree species’ habitat suitability shifts under future conditions, we estimated two spatial metrics. The first metric was estimated as the per cent change of future to the current area of species’ habitat suitability and indicates the degree of “habitat availability”, i.e., whether the areas with favourable conditions increase (+) or decrease (−) under future conditions. The second metric was the difference in mean elevation between the current and future habitat suitability. The larger the difference, the higher the distance populations of the species would need to travel to achieve favourable conditions. Dispersal limitations due to anthropogenic activities such as barriers or land-use changes were not considered. All analyses and maps were made with the R programming language [124]. 

## 5. Conclusions

In this study, we modelled the current and future habitat availability of the dominant tree species in Greece. Overall, species currently found in lower elevation Mediterranean forests, such as *P. brutia*, *P. halepensis* and *Q. ilex,* were projected to suffer smaller suitable habitat area losses compared to mountainous taxa, such as *P. nigra, Q. pubescens, Q. frainet*-to and *F. sylvatica*. Under most climate change scenarios, the two Mediterranean fir species of our study (*A. cephalonica* and *A. borisii-regis*) presented a rather stable total suitable area which, if valid, could promote their potential use in climate mitigation policies. Although other important taxa found at both low and mid-elevations were not included in this study, our findings might be useful for inferring the vulnerability of the forest sector in Greece and its potential for adaptation under warmer future conditions.

## Figures and Tables

**Table 1:** Summary of the final maximum entropy model for the distribution of the studied species. The relative contribution of each environmental variable to the final model of each species is also presented, with T_max_: mean daily maximum air temperature of the warmest month (°C), T_min_: mean daily minimum air temperature of the coldest month (°C), P_a_: total annual precipitation (mm), P_dm_: precipitation of the driest month (mm), GDD5: heat sum of all days above 5 °C accumulated over a year (°C), GSL: length of the growing season (days), GST: mean temperature of all growing season days (°C), GSP: precipitation accumulated during the growing season (mm), parmat: dominant parent material class and texture: dominant surface textural class.

Environmental Variables	*Pinus halepensis*	*Pinus brutia*	*Pinus nigra*	*Abies* spp.	*Quercus ilex*	*Quercus pubescens*	*Quercus frainetto*	*Fagus sylvatica*
T_max_	47.4				41.9	49.2		29.9
T_min_				12.2				
P_a_						7.6		12.4
P_dm_	15.0	26.5	8.7	19.8	24.3		28.4	
GDD5		37.2	35.8				37.9	
GSL		8.7			13.6	16.3		36.2
GST			6.5		7.3		8.5	13.4
GSP			18.8	9.0			10.2	
parmat	37.6	9.1	30.3	12.8	12.8	26.9	15.0	8.1
texture		18.5		46.2				
# occurrences (thinned)	1181	38	2041	67	2455	1896	209	6634
TSS	0.80	0.91	0.58	0.81	0.78	0.66	0.77	0.61
AUC	0.95	0.98	0.86	0.94	0.94	0.90	0.96	0.86

**Table 2:** Projected changes in habitat availability (% of the current suitable area) and mean elevation shift (m between current and future climate) under the two scenarios SSP1-2.6 and SSP5-8.5 for the two reference periods 2041–2071 and 2071–2100 for each studied species.

Change of Habitat Availability (%)	*Pinus halepensis*	*Pinus brutia*	*Pinus nigra*	*Abies* spp.	*Quercus ilex*	*Quercus pubescens*	*Quercus frainetto*	*Fagus sylvatica*
SSP1-2.6_2070	−8	−17	−38	+17	−14	−16	−28	−56
SSP1-2.6_2100	−6	−14	−36	+25	−1	−24	−28	−60
SSP5-8.5_2070	−21	−32	−53	0	−18	−42	−44	−75
SSP5-8.5_2100	−45	−54	−77	−27	−47	−64	−72	−93
**Shift in Mean** **Elevation (m)**	***Pinus halepensis***	***Pinus brutia***	***Pinus nigra***	***Abies* spp.**	***Quercus ilex***	***Quercus pubescens***	***Quercus frainetto***	***Fagus sylvatica***
SSP1-2.6_2070	+139	+175	+257	-3	+95	+143	+233	+285
SSP1-2.6_2100	+159	+164	+233	−13	+71	+159	+253	+293
SSP5-8.5_2070	+209	+236	+359	+63	+195	+262	+375	+434
SSP5-8.5_2100	+330	+333	+599	+185	+387	+307	+650	+655

## Data Availability

The maps of the current and future habitat suitability are available in GeoTIFF format as Supplementary Materials.

## Supplementary Materials

The following supporting information can be downloaded at: https://www.mdpi.com/article/10.3390/plants11121616/s1. GeoTIFF files for Figure 3, Figure 4, Figure 5, Figure 6, Figure A2 and Figure A7 are provided in Supplementary Files S1–S5.
